# Editorial: Non-ionizing radiation: impacts on human health from exposures in occupational settings

**DOI:** 10.3389/fpubh.2026.1880564

**Published:** 2026-07-13

**Authors:** Alberto Modenese, Claudina M. C. A. Nogueira, Marc Wittlich

**Affiliations:** 1 Department of Biomedical Metabolic and Neural Sciences, University of Modena and Reggio Emilia, Modena, Italy; 2 Department of Global Health, Faculty of Medicine and Health Sciences, Stellenbosch University, Parow, Western Cape, South Africa; 3 Institute for Occupational Safety and Health, German Social Accident Insurance (IFA), Sankt Augustin, Germany

**Keywords:** electromagnetic fields (EMFs), exposure measurement, non-ionizing radiation, occupational exposure, optical radiation, risk assessment, ultraviolet (UV) exposure, worker protection

Non-ionizing radiation (NIR) is an increasingly prevalent feature of modern workplaces, spanning sources from power-frequency electromagnetic fields (EMFs) and radiofrequency emissions to optical radiation used in industry, healthcare, telecommunications, and advanced manufacturing systems. The rapid deployment of 5G/6G communication infrastructure, Industry 4.0 environments characterized by interconnected wireless devices and automated systems, and the growing use of laser-based and optical clinical technologies are contributing to increasingly complex occupational exposure scenarios. As technologies evolve and exposure profiles diversify, so too does the evidence base—often progressing alongside persistent scientific uncertainty regarding long-term and low-level effects. This Research Topic brings together current research and perspectives to examine occupational exposures to NIR across sectors, critically appraise emerging evidence, and reflect on the implications for risk assessment, standards, and worker protection. By linking evidence, uncertainty, and prevention, the collection seeks to inform more discerning, protective, and context-responsive approaches to safeguarding worker health in evolving exposure scenarios.

NIR encompasses optical radiation—ultraviolet (UV), visible light, and infrared radiation—as well as EMFs ranging from static electric and magnetic fields to extremely high-frequency fields up to 300 GHz. Both optical radiation and EMFs are widely present in contemporary occupational environments. In addition, solar optical radiation represents a well-recognized hazard for occupations that require prolonged work outdoors (1–7).

The Research Topic, “*Non-ionizing radiation: impacts on human health from exposures in occupational settings*” was conceived to address persistent knowledge gaps and to foster interdisciplinary dialogue on the health implications of occupational exposures to NIR. The collection aims to (i) advance understanding of

biological mechanisms and health outcomes associated with NIR, (ii) evaluate exposure scenarios in real-world occupational contexts, and (iii) identify effective preventive and protective strategies to reduce risk and enhance worker safety. These objectives are particularly timely given the rapid technological evolution driving both the increasing diversity and intensity of occupational exposures to NIR.

The 10 papers included in this Research Topic collectively reflect the multifaceted nature of NIR research, spanning experimental, epidemiological, and applied perspectives. Taken together, the contributions consistently highlight a central challenge in occupational NIR protection: workplace exposures are often highly dynamic, task-dependent, and insufficiently characterized by simplified or time-averaged metrics alone. Despite addressing different exposure agents, occupational sectors, and methodological approaches, the studies converge in showing that exposure variability itself is a key determinant of risk characterization. A recurring finding across both EMF and optical radiation investigations is that worker-specific activities, environmental conditions, and source configurations can substantially influence actual exposure patterns, supporting a shift toward more individualized and task-oriented assessment strategies. Across optical radiation and EMF scenarios, the studies collectively support the need for increasingly context-specific exposure assessment approaches capable of capturing spatial and temporal variability in real working environments.

Several contributions focus on UV radiation, a well-characterized component of NIR with clear occupational relevance, while also emphasizing practical prevention. For example, the study of Al-Hadyan et al. on personal protective equipment (PPE) shows that materials commonly used for respiratory protection or infection control may provide variable—and sometimes insufficient—UV attenuation, depending on wavelength and fabric properties. This finding is particularly relevant in post-pandemic occupational settings, where PPE use has expanded but is rarely assessed from a radiation protection perspective. Together, these findings suggest that incorporating UV protection criteria into PPE design and selection could represent a simple yet impactful preventive measure.

Other papers address radiofrequency and low-frequency EMFs, increasingly prevalent due to the expansion of wireless technologies, smart industrial systems, and advanced healthcare applications. A key contribution of this work is improved exposure characterization: the occupational measurement and modeling studies included in the collection demonstrate that field intensities can vary substantially, depending on proximity to sources, task duration, body position, shielding conditions, and simultaneous operation of multiple emitting systems. Occupational settings such as telecommunications, healthcare, and industrial environments may therefore present heterogeneous exposure patterns, sometimes revealing localized short-duration peaks not adequately represented by conventional averaged exposure estimates. Collectively, these findings raise important questions regarding the practical implementation of current exposure assessment strategies based on available recommendations, such as International Commission on Non-Ionizing Radiation Protection (ICNIRP) guidelines (1–4), particularly in relation to task-based and highly localized exposures in complex workplaces and to the possible presence of workers with conditions of higher susceptibility to the risk, also considering indirect effects of NIR exposure. These considerations are particularly relevant in emerging occupational environments associated with Industry 4.0 processes, the Industrial Internet of Things (IIoT), artificial intelligence-driven automation, smart manufacturing systems, interconnected wireless infrastructures, and the deployment of current and future 5G/6G communication networks. In these environments, workers may be exposed to increasingly complex and heterogeneous EMFs arising from the simultaneous operation of multiple NIR sources. Unlike traditional occupational settings where exposures often originate from a limited number of identifiable sources, modern workplaces are characterized by dynamic and rapidly changing exposure scenarios that vary spatially and temporally according to network traffic, device density, operational demands, and worker mobility.

These evolving exposure patterns present new challenges for occupational exposure assessment and risk management, particularly as higher-frequency millimeter-wave and quasi-millimeter-wave technologies become increasingly prevalent. Conventional monitoring approaches may not adequately capture the complex and dynamic exposures associated with emerging NIR environments. In this context, the experimental study by Ijima et al., which investigated biological effects in a rat model exposed to quasi-millimeter waves, contributes valuable evidence to the growing body of research on higher-frequency NIR exposures. Although occupational exposures are generally expected to remain below established safety limits, the increasing adoption of Industry 4.0 technologies—including smart factories, private industrial 5G/6G networks (in manufacturing, mining, logistics, ports, and energy sectors), wireless power transfer systems, and inductive charging stations for autonomous vehicles and robotics—highlights the need for ongoing surveillance, improved exposure assessment methodologies, worker education, and continued research to ensure that occupational health protection frameworks remain aligned with technological advances and support evidence-based prevention in future workplaces.

Peyró-Sánchez et al. characterized ambient exposure to radiofrequency EMFs from telecommunication antennas and Wi-Fi sources during surgical operations in hospital operating rooms and concluded that ambient exposure remained below 0.4% of the ICNIRP reference level.

Colella et al. examined EMF exposure from military vehicular antennas, covering a broad spectrum of frequencies, power levels, and positions. Their findings suggest that personnel safety in military contexts is generally maintained, even in the presence of variable exposure conditions and elevated levels of radiated electric fields.

Interestingly, despite addressing substantially different occupational environments, both the healthcare scenario investigated by Peyró-Sánchez et al. and the military applications examined by Colella et al. indicate that detailed exposure characterization is essential for accurately assessing worker risk. Together, these studies suggest that the principal challenge is often not the exceedance of exposure limits, but rather the appropriate characterization of complex and highly variable exposure conditions.

In terms of health effects, experimental or observational studies in the collection explore endpoints such as oxidative stress markers, neurocognitive outcomes, and subjective symptoms, contributing to an evidence base that is still evolving and not always consistent. In addition, potential indirect risks warrant consideration, particularly for workers with specific susceptibilities related to active implanted or wearable medical devices. These aspects should be appropriately addressed within occupational health surveillance programs.

The study by Gasparini et al. provides a solid foundation for quantifying the actual impact of workers with active implantable and active wearable medical devices, as these workers are considered to require occupational risk assessment and health surveillance procedures, due to possible electromagnetic hazards resulting in interference issues.

Ijima et al. evaluated histological changes and the expression of inflammation-related markers in rat skin tissue locally exposed to quasi-millimeter waves and investigated the threshold for inflammatory responses. Although the study was conducted in a rat model, their findings suggest that current guidelines appear to ensure the safety of human organs and tissues exposed to quasi-millimeter waves, as used in modern 5G networks.

A further strength of this Research Topic lies in its attention to methodological and preventive contributions. Several papers highlight advances in dosimetry, including wearable sensors and task-based exposure assessment approaches that enable more refined individual exposure profiling. Others emphasize the need to harmonize measurement protocols and align occupational exposure assessment with international guidelines, highlighting gaps between regulatory limits and real-world monitoring practices. Importantly, the collection suggests that future occupational NIR risk management may increasingly require integrated monitoring strategies capable of accounting for intermittent, source-specific, and worker-specific exposure determinants rather than relying exclusively on generalized environmental measurements. Moreover, not only artificial sources need to be carefully assessed; carcinogenic solar UV radiation exposure needs to be evaluated in outdoor workplaces. These methodological developments are essential for improving comparability across studies and strengthening evidence-based risk management.

Bieck et al. gathered data about national regulations on the use of tanning beds and worker's protection from solar UV radiation, since skin cancer is a rapidly increasing global public health concern. Their findings show that action is needed in terms of skin cancer prevention on different levels, and particularly in high-risk groups such as outdoor workers and in the general population with limited awareness or understanding of UV radiation exposure risks, as evidenced through tanning bed use.

de Boer et al. evaluated the short-term effectiveness of a sun-safety risk communication toolbox aimed to increase sun-safety behavior among male outdoor workers. The toolbox intervention led to a reduction of internal UV radiation exposure, consistent with a self-reported increase in sunscreen use, compared to no intervention.

Taken together, the UV-related studies included in this Research Topic underscore the importance of adopting a holistic prevention framework that extends beyond traditional exposure-control measures. While engineering controls and personal protective measures remain indispensable for mitigating occupational UV exposure, the findings suggest that behavioral interventions, risk communication, and health promotion initiatives can play a critical complementary role in influencing exposure-related behaviors and enhancing adherence to protective practices. The integration of technical and behavioral approaches may therefore offer greater potential for achieving sustained reductions in occupational UV exposure and associated health risks than either strategy alone. These findings reinforce the need for multifaceted prevention programs that address not only the physical determinants of exposure but also the knowledge, attitudes, perceptions, and organizational factors that influence worker behavior and long-term prevention outcomes.

The Research Topic also highlights the importance of distinguishing between beneficial patient-directed uses of NIR technologies and occupational risks affecting exposed operators. In healthcare settings, technologies such as medical lasers, phototherapy systems, and radiofrequency-based devices may provide important clinical benefits for patients while simultaneously generating occupational exposure scenarios for healthcare workers responsible for operating or maintaining such systems. This distinction reinforces the need for exposure-specific occupational prevention measures even in environments where NIR technologies are primarily used for therapeutic or diagnostic purposes. Accordingly, occupational risk assessment should remain distinct from clinical benefit evaluation, ensuring that patient outcomes and worker protection are addressed through complementary but separate preventive frameworks.

When considered collectively, the contributions to this Research Topic provide a more detailed view of occupational exposures to NIR than is typically available, highlighting quantifiable risks alongside opportunities for prevention. Beyond the specific findings of individual studies, the collection underscores the broader need for adaptive occupational radiation protection frameworks capable of responding to emerging technologies, natural sources not properly addressed, increasingly complex exposure environments, and persistent scientific uncertainty regarding long-term low-level effects.

The defining challenge of modern occupational health is no longer the mere exceedance of exposure limits, but rather the unprecedented spatial and temporal variability inherent in digitalized workplaces. This Research Topic demonstrates that as we transition into Industry 4.0 and 5G/6G environments, conventional time-averaged metrics fail to capture the dynamic reality of NIR exposure, necessitating a fundamental shift toward task-oriented, individualized assessment strategies and holistic, behavior-driven prevention frameworks.

As Guest Editors, we hope this Research Topic will stimulate further research, inform policy development, and ultimately contribute to safer occupational environments in an increasingly technology-driven world. Addressing the challenges posed by NIR requires not only scientific rigor but also interdisciplinary collaboration and an initiative-taking approach to worker protection.

## References

[1] International Commission on Non-Ionizing Radiation Protection (ICNIRP). Guidelines for limiting exposure to time-varying electric and magnetic fields (1 Hz−100 kHz). Health Phys. (2010) 99:818–36. doi: 10.1097/HP.0b013e3181f06c86 21068601

[2] International Commission on Non-Ionizing Radiation Protection (ICNIRP). Guidelines on limits of exposure to incoherent visible and infrared radiation. Health Phys. (2013) 105:74–96. doi: 10.1097/HP.0b013e318289a611 35606999

[3] International Commission on Non-Ionizing Radiation Protection (ICNIRP). ICNIRP statement on the principles of non-ionizing radiation protection. Health Phys. (2020) 118:477–82. doi: 10.1097/HP.0000000000001252 32251080

[4] International Commission on Non-Ionizing Radiation Protection (ICNIRP). Guidelines for limiting exposure to electromagnetic fields (100 kHz to 300 GHz). Health Phys. (2020) 118:483–524. doi: 10.1097/HP.0000000000001210 32167495

[5] International Labour Organization (ILO). Safety and Health in the Use of Optical Radiation in the Workplace. Geneva: International Labour Office (2017).

[6] World Health Organization (WHO). Environmental Health Criteria 238: Extremely Low Frequency Fields. Geneva: World Health Organization (2007).

[7] World Health Organization and and International Agency for Research on Cancer (IARC). Solar and Ultraviolet Radiation. IARC Monographs on the Evaluation of Carcinogenic Risks to Humans, Vol. 100D. Lyon: International Agency for Research on Cancer (2012).

